# Multi-Mode Imaging Scale for Endovascular Therapy in Patients with Acute Ischemic Stroke (META)

**DOI:** 10.3390/brainsci12070821

**Published:** 2022-06-24

**Authors:** Wansi Zhong, Zhicai Chen, Shenqiang Yan, Ying Zhou, Ruoxia Zhang, Zhongyu Luo, Jun Yu, Min Lou

**Affiliations:** 1 Department of Neurology, The Second Affiliated Hospital of Zhejiang University, School of Medicine, Hangzhou 310009, China; 21718233@zju.edu.cn (W.Z.); chenzhicai@zju.edu.cn (Z.C.); shenqiangyan@zju.edu.cn (S.Y.); zh_ying@zju.edu.cn (Y.Z.); 0620548@zju.edu.cn (R.Z.); 11818065@zju.edu.cn (Z.L.); 2 Department of Neurosurgery, The Second Affiliated Hospital of Zhejiang University, School of Medicine, Hangzhou 310009, China; 2505020@zju.edu.cn

**Keywords:** acute ischemic stroke, endovascular thrombectomy, multi-mode imaging, outcome

## Abstract

Background: With the guidance of multi-mode imaging, the time window for endovascular thrombectomy (EVT) has been expanded to 24 h. However, poor clinical outcomes are still not uncommon. We aimed to develop a multi-mode imaging scale for endovascular therapy in patients with acute ischemic stroke (META) to predict the neurological outcome in patients receiving endovascular thrombectomy (EVT). Methods: We included consecutive acute ischemic stroke patients with occlusion of middle cerebral artery and/or internal carotid artery who underwent EVT. Poor outcome was defined as modified Rankin Scale (mRS) score of 3–6 at 3 months. A five-point META score was constructed based on clot burden score, multi-segment clot, the Alberta Stroke Program early computed tomography score of cerebral blood volume (CBV-ASPECTS), and collateral status. We evaluated the META score performance using area under the curve (AUC) calculations. Results: A total of 259 patients were included. A higher META score was independently correlated with poor outcomes at 3 months (odds ratio, 1.690, 95% CI, 1.340 to 2.132, *p* < 0.001) after adjusting for age, hypertension, baseline National Institutes of Health Stroke Scale (NIHSS) score, and baseline blood glucose. Patients with a META score ≥ 2 were less likely to benefit from EVT (mRS 3–6: 60.8% vs. 29.2%, *p* < 0.001). The META score predicted poor outcomes with an AUC of 0.714, higher than the Pittsburgh Response to Endovascular therapy (PRE) score, the totaled health risks in vascular events (THRIVE) score (AUC: 0.566, 0.706), and the single imaging marker in the scale. Conclusions: The novel META score could refine the predictive accuracy of prognosis after EVT, which might provide a promising avenue for future automatic imaging analysis to help decision making.

## 1. Introduction

Endovascular thrombectomy (EVT) has been proved to be the most effective treatment in acute ischemic stroke (AIS) patients with large vessel occlusion [1]. However, there is still a large number of patients who achieve poor outcome despite successful recanalization after EVT, suggesting that a better patient selection strategy is needed [2,3,4,5]. The decision-making process for EVT is critical in maximizing the clinical benefit. Selection of patients for EVT using advanced multi-mode imaging assists in increasing diagnostic accuracy, quantification of tissue at risk, and prognostication of functional outcome. Subsequently, advanced multi-mode imaging has been administered to select eligible patients for thrombolysis and EVT [6]. A systematic review and meta-analysis of eight observational studies (*n* = 2813) revealed that patient selection using perfusion imaging in AIS increased the probability of three-month functional independence by two-fold [7]. Other imaging markers such as thrombus permeability, clot burden score (CBS), deep cerebral vein, and collaterals were all reported to be associated with functional independence after EVT [8,9,10,11].

Most previous studies have only focused on one certain imaging marker to predict the clinical functional outcome after EVT [12,13]; however, no single marker can fully reflect the whole pathophysiological information of ischemia. This leads to a question of whether an integrated assessment scale can better reflect ischemic status and predict prognosis after EVT in large vessel occlusion. In the current study, we thus developed a multi-mode prognostic model: multi-mode imaging scale for endovascular therapy in patients with acute ischemic stroke (META), and hypothesized that META could refine the predictive accuracy of prognosis after EVT. Binary logistic regression analyses were used to select the imaging parameter into the META score.

## 2. Materials and Methods

### 2.1. Subject

We retrospectively reviewed our prospectively collected database for consecutive AIS patients who received EVT between October 2013 and October 2020. This study was approved by the human ethics committee of our center. The clinical investigation was conducted according to the principle expressed in the Declaration of Helsinki. Written informed consent was obtained from all patients.

We enrolled patients who (1) had a diagnosis of AIS, (2) had occlusion in the middle cerebral artery and/or internal carotid artery and were treated by EVT, and (3) underwent computed tomography perfusion (CTP) before EVT. Patients (1) with pre-stroke modified Rankin Scale (mRS) score ≥ 2, (2) without three-month mRS, or (3) who had incomplete CTP raw images or poor-quality reconstructed images were excluded.

### 2.2. Demographics, Variables and Measurements

Baseline variables including demographics, risk factors (history of hypertension, diabetes, stroke or TIA, or atrial fibrillation), prior antiplatelet usage, onset to door time (ODT), baseline National Institutes of Health Stroke Scale (NIHSS) score, baseline blood glucose, baseline infarct core volume, and status of reperfusion at 24 h were recorded. Clinical outcome at 3 months was dichotomized into good outcome (mRS 0–2) and poor outcome (mRS 3–6).

We then compared the prognostic value of the META with previously established prediction scales including Pittsburgh Response to Endovascular therapy (PRE) score and totaled health risks in vascular events (THRIVE) score, as previously described [13,14].

### 2.3. Imaging Protocol and Image Analysis

CTP was performed on a dual-source 64-slice CT scanner (SOMATOM Definition Flash; Siemens, Forchheim, Germany), including non-enhanced computed tomography (NECT) head scan (120 kV, 320 mA, contiguous 5 mm axial slices), and volume perfusion CT (100 mm in the *z*-axis, 4 s delay after start of contrast medium injection, 74.5 s total imaging duration, 80 kV, 120 mA, effective dose = 3.68 mSv, slice thickness 10 mm, collimation 32 × 1.2 mm). Volume CTP consisted of 26 consecutive spiral acquisition of the brain. All 26 scans were divided into 4 parts: (1) 2 scans with 3 s cycle time; (2) 15 scans with 1.5 s cycle time; (3) 4 scans with 3 s cycle time; and (4) 5 scans with 6 s cycle time. Axial slice coverage was 150 mm. A 60 mL bolus of contrast medium (Iopamidol; Braccosine, Shanghai, China) was used at a flow rate of 6 mL/s, followed by a 20 mL saline chaser at 6 mL/s.

Infarct core was defined as relative cerebral blood flow (rCBF) < 30% [15]. The presence of hypoperfusion of lenticulostriate artery (LSA) territory (marked as: LSA-) was defined as hypoperfusion in LSA territory but not in the terminal branch territory of the middle cerebral artery [16]. We evaluated the extent of lesions on NECT, CBF, and cerebral blood volume (CBV) imaging using the Alberta Stroke Program early computed tomography score (ASPECTS) methodology, described by Barber et al. [17]. We reviewed the NECT, CBV, and CBF maps and qualitatively evaluated NECT-ASPECTS, CBV-ASPECTS, and CBF-ASPECTS [18]. CBS is a scoring system to define the extent of thrombus located in the proximal anterior circulation and is scored on a scale of 0–10 [18]. Poor collateral was defined as no vessel marking or less than half of the middle cerebral artery territory in the ischemic side [19]. The multi-segment clot (MSC) sign was defined as more than one complete filling defect on dynamic computed tomographic angiography derived from CTP [20]. Two experienced neurologists blinded to the patients’ information assessed these imaging makers, with rater discrepancies settled by consensus discussion.

### 2.4. Statistical Analysis

Statistical analysis was performed using SPSS 22.0 (SPSS, Inc., Chicago, IL, USA). Clinical characteristics were summarized as mean ± standard deviation (SD) or median (25th–75th percentile) for quantitative variables and as proportions for categorical variables. Fisher’s exact test was used to compare the dichotomous variables, whilst the independent samples two-tailed t-test or Mann–Whitney U test was used for the continuous variables, as appropriate. The continuous variables with *p* values < 0.05 in univariate analysis between poor outcome and good outcome were dichotomized according to the optimal cutoff derived from the receiver operating characteristic (ROC) curve. Binary logistic regression analyses were performed to identify independent predictors of poor outcome after adjusting for potential confounding factors. Each imaging parameter was entered in the binary logistic regression models alone as the potential collinearity among these imaging parameters. Independent predictors with *p* < 0.05 entered final META score. The points assigned to the variables of the META score were determined through the OR from the binary logistic regression models. The Hosmer and Lemeshow statistic was used to determine the sensitivity and specificity of the model and to assess goodness of fit. Model calibration was assessed with a calibration curve (*p* > 0.05 was considered good calibration). ROC area under the curve (AUC) was used to assess the discriminative power of META for poor outcome. A *p* value of < 0.05 was considered to be statistically significant.

## 3. Results

Among 325 patients meeting inclusion criteria, 28 patients with mRS ≥ 2 prior to the index stroke, 23 patients without three-month mRS, and 15 patients without complete CTP raw images or poor-quality reconstructed images were excluded. The final analysis includes 259 patients. Of the included patients, the mean age was 69 ± 12 years and 101 (39.0%) were women. Baseline NIHSS was 14 (11–18), median ODT was 206 (98–333) min, and 153 (59.1%) had a poor outcome at 3 months.

As Table 1 shows, patients with a poor outcome were older (*p* < 0.001), had higher baseline NIHSS (*p* < 0.001), higher baseline blood glucose (*p* < 0.001), and higher rate of hypertension (68.0% versus 54.7%, *p* = 0.037) than those with a good outcome. In contrast, the sex, ODT, and other stroke risk factors were comparable between the two group. Moreover, imaging markers including CBS, CBV-ASPECTS, CBF-ASPECTS, baseline core volume, NECT-ASPECTS, LSA-, poor collateral, absence of MSC, and baseline occlusion location were significantly different.

### 3.1. Development of Multi-Mode Prognostic Algorithm META

ROC analysis showed that the optimal cutoff value of CBS, CBV-ASPECTS, and NECT-ASPECTS in predicting a poor outcome was all 6, while CBF-ASPECTS was 3 and baseline core volume was 55 mL. As Table 2 shows, binary logistic regression analyses revealed that CBS ≤ 6, CBV-ASPECTS ≤ 6, NECT-ASPECTS ≤ 6, baseline core volume ≥ 55 mL, absence of MSC, and poor collateral were independent predictors of poor outcome after adjusting for age, hypertension, baseline NIHSS score, and baseline blood glucose. CBV-ASPECTS, instead of baseline core volume and NECT-ASPECTS, was selected because it performed better (Table 2). We thus included CBS, CBV-ASPECTS, MSC, and collateral in the final META score (Table 3).

A META score of 0, 1, 2, 3, 4 and 5 was assigned to 39 (15.1%), 96 (37.1%), 40 (15.4%), 30 (11.6%), 36 (13.9%), and 18 (6.9%) patients, respectively. Calibration of the regression model was satisfactory (Hosmer–Lemeshow test *p* = 0.65).

### 3.2. Association of META Score with Outcome

**Figure 1:**
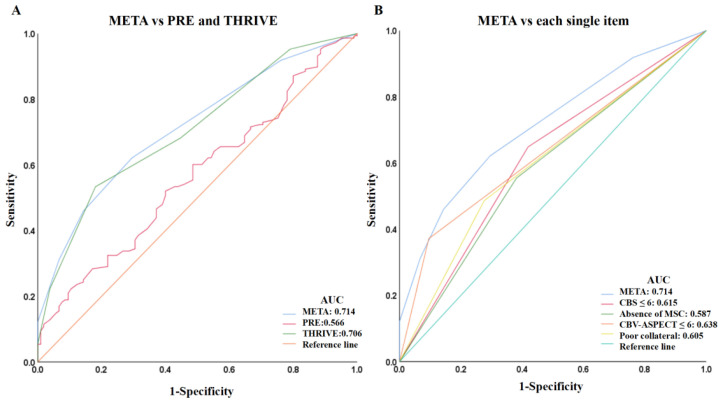
ROC analysis for poor outcome. (**A**), Discriminative power (area under the curve (AUC)) of META, PRE, and THRIVE for poor outcome; (**B**), AUC of META and each single item of META score for poor outcome.

Binary logistic regression showed that the META score was independently correlated with a poor outcome (OR = 1.690, 95% CI, 1.340 to 2.132, *p* < 0.001) after adjusting for age, baseline NIHSS score, baseline blood glucose, and hypertension. The ROC curve of the META score for predicting a poor outcome is shown in Figure 1, and the optimal cutoff was 3 (AUC 0.714; *p* < 0.001). The specificity and sensitivity of the META score for predicting a poor outcome were 71% and 62%, respectively. Patients with a META score ≥ 2 had about a two-fold rate of a poor outcome (60.8% vs. 29.2%, *p* < 0.001) than those with a META score 0–1. All 18 (6.9%) patients with a META score of 5 showed poor outcomes at 3 months.

**Figure 2:**
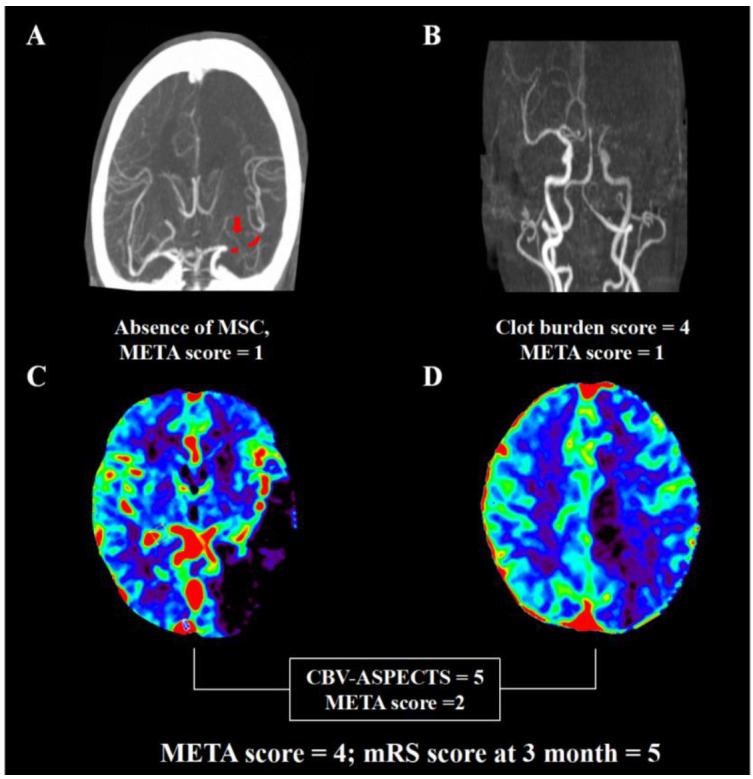
Representative images for calculation of META. A male patient had occlusion of left middle cerebral artery on dynamic computed tomography angiography (**A**,**B**). He had clot burden score of 4 (occlusion of M1 and M2), one segment of clot (red dot that the red arrow points to, (**A**)), good collateral with >50% filling of the occluded middle cerebral artery territory (**A**). Cerebral blood volume map (**C**,**D**) demonstrated the Alberta Stroke Program early CT score of 5. Thus, the META score was 4. The modified Rankin score was 5 at 3 months despite successful recanalization.

The AUC of the META score was higher than other previously established prediction scales including the PRE score, THRIVE score, and any single component in the META score (Figure 1). The risk of poor outcome increased with a higher META score (0 = 35.9%, 1 = 47.9%, 2 = 60.0%, 3 = 73.3%, 4 = 80.6%, and 5 = 100%; *p* < 0.001). Representative images of multi-mode imaging in META are shown in Figure 2.

## 4. Discussion

We piloted a novel META score based on multiple imaging features in AIS patients receiving EVT. Increasing META score was associated with a higher likelihood of poor outcomes at 3 months.

The novel META score performed better in predicting the clinical outcome after EVT than previous predictive scales, including the PRE score (including age, NIHSS, and ASPECT) and THRIVE score (including age, NIHSS, diabetes mellitus, hypertension, and atrial fibrillation) [13,14,21]. Both of them include age and NIHSS, and tend to exclude older or severer patients from EVT to some extent, which is not reasonable. The Highly Effective Reperfusion Evaluated in Multiple Endovascular Stroke Trials (HERMES) meta-analysis of five randomized controlled trials demonstrated that EVT patients aged 80 years and older still had a higher rate of good outcomes compared with those without EVT [22]. Moreover, EVT could also improve outcomes in patients with severe stroke [23]. Based on this evidence, EVT is still recommended by current guidelines for patients older than 80 years or with severe symptoms [24]. In view of this potential risk for a part of patients to lose the opportunity of EVT, we only included imaging markers in the META score. Accumulating evidence has shown that advanced imaging is more crucial than clinical characteristics for the prognostic prediction of EVT [25] by giving an insight into the pathophysiological mechanisms.

All imaging markers included in the META score have been proven to predict the neurological outcome of AIS patients in previous studies [9,10,19,20,26]. (1) Lower CBS was associated with a lower likelihood of recanalization and unfavorable functional outcomes, as the proximal and long clot was harder to treat, leading to a worse outcome compared with the short distal clot [27]. On the other hand, the MSC sign, indicating the clot fragmentation, was related to a higher rate of recanalization and good outcomes after reperfusion therapy. (2) Perfusion CBV, reflecting the compensation for ischemia, was better at predicting clinical outcomes than NECT, which is similar to our results [17]. (3) Collaterals have been widely confirmed as a predictor of prognosis and recanalization after reperfusion therapy. Hence, each imaging marker, from clot and arterial collaterals to ischemic tissue, displays the ischemia-related pathophysiological status, and thus jointly influences vascular recanalization and tissue destiny after cerebral ischemia. The application of a comprehensive score may be more practical and objective to offer a precise prediction in AIS patients.

Multi-mode imaging is increasingly used to select patients who would benefit from EVT beyond 6 h after stroke onset after the results of DAWN and DEFUSE 3 trials were published [28,29]. However, real-world data actually revealed a lower rate of good clinical outcomes at 3 months than that reported in HERMES (37% vs. 46%) [30]. The adjuvant identification of large vessel occlusion patients with a potential high risk of poor outcomes, based on neuroimaging findings, may improve the decision making of clinicians in clinical practice. The META score may also be useful in patient selection for EVT across different cohorts in clinical trials.

Clinicians may be concerned about the time consumption of processing and assessment of image data. Artificial intelligence (AI) technology, which is a rapidly burgeoning field, can provide fast and efficient automatic imaging analysis [31]. Through machine learning, a recent study demonstrated that CTP data could be used to estimate follow-up infarct in AIS patients [32]. The existing commercial software RAPID can automatically calculate the ASPECTS in AIS patients [33]. Moreover, machine learning algorithms also provide a new idea for the automatic prediction of neurological outcomes in AIS patients who underwent EVT [34]. Based on the META score, which only includes imaging markers, automatic detection of these imaging markers via AI may assist with improving the accuracy of imaging evaluation and decreasing the time consumption of clinicians. Thus, outcome prediction software based on the META score can be designed for simple and fast clinical application. Nevertheless, different imaging markers were assessed based on different imaging modalities, which may influence model performance of machine learning.

There are some limitations in our study. Firstly, although we prospectively collected data using a stroke registry, our study had a retrospective design and might have a potential risk of selection bias. With the notable improvement of interventional vascular neurology and devices, it is possible that recanalization is improved and more patients may benefit. Secondly, the current study only proved that the prognosis was worse in those with a higher META score, but whether EVT should be given to those with a high META score is still unknown. Overall, further randomized controlled trials are needed to confirm the validity of the META score. Thirdly, although this study confirmed the effect of the META score on poor outcomes, both sensitivity and specificity were modest. It should be kept in mind that a considerable proportion of patients with a META score of 3 or above nevertheless have a good prognosis.

## 5. Conclusions

The META score, a comprehensive score based on multi-modal images, was associated with neurological outcomes and performed better than previous established scales. It may assist clinicians in decision making for EVT in anterior circulation large artery occlusion. Further automatic outcome prediction software based on the META score might be designed for EVT patient selection and prognostic prediction in the future.

## Figures and Tables

**Table 1:** Comparison between poor outcome and good outcome group.

Variables	Poor Outcome (*n* = 153)	Good Outcome (*n* = 106)	*p* Value
**Clinical characters**			
Age, years	71 ± 12	65 ± 13	<0.001
Women, (%)	58 (37.9)	43 (40.6)	0.699
Baseline NIHSS, IQR	16 (12–18)	12 (9–16)	<0.001
Onset to door time, min, IQR	211 (110–316)	178 (96–352)	0.473
Intravenous thrombosis, (%)	106 (69.3)	81 (76.4)	0.259
Baseline blood glucose, mg/dL, IQR	126 (115–153)	121 (108–139)	0.008
**Radiological data**			
Clot burden score, IQR	6 (4–8)	7 (5–8)	0.017
CBV-ASPECTS, IQR	8 (5–9)	8 (8–9)	<0.001
CBF-ASPECTS, IQR	4 (2–6)	5 (3–6)	<0.001
Baseline core volume, mL, IQR	40 (21–72)	28 (17–46)	0.001
NECT-ASPECTS, IQR	8 (6–9)	9 (7–9)	0.006
Presence of LSA-, (%)	112 (73.2)	64 (60.4)	0.031
Poor collateral, (%)	73 (47.7)	29 (27.4)	0.001
Absence of MSC, (%)	83 (54.2)	41 (38.7)	0.016
Baseline occlusion location, (%)			0.045
Internal carotid artery	69 (45.1)	32 (30.2)	
Middle cerebral artery-M1	63 (41.2)	58 (54.7)	
Middle cerebral artery-M2	21 (13.7)	16 (15.1)	
**Risk factors**			
Hypertension, (%)	104 (68.0)	58 (54.7)	0.037
Diabetes, (%)	30 (19.6)	11 (10.4)	0.056
Prior antiplatelet usage, (%)	27 (17.6)	20 (18.9)	0.870
History of stroke/TIA, (%)	27 (17.6)	18 (17.0)	1.000
Atrial fibrillation, (%)	80 (52.3)	50 (47.2)	0.450

Value are mean ± SD, median (interquartile range), or No. (%) as appropriate. NIHSS, National Institutes of Health Stroke Scale; IQR, Interquartile Range; CBV, Cerebral Blood Volume; ASPECTS, the Alberta Stroke Program Early Computed Tomography Score; CBF, Cerebral Blood Flow; NECT, Non-Enhanced Computed Tomography; LSA, Lenticulostriate Arteries; MSC, Multi-Segment Clot.

**Table 2:** Logistic regression analysis for poor outcome.

Variables	OR	95% CI	*p* Value
CBS ≤ 6	1.928	1.083–3.432	0.027
CBV-ASPECTS ≤ 6	3.873	1.751–8.563	0.001
CBF-ASPECTS ≤ 3	1.612	0.907–2.865	0.104
NECT-ASPECTS	2.395	1.180–4.861	0.016
Baseline core volume ≥ 55 mL	2.014	1.015–3.996	0.045
Presence of LSA-	1.400	0.773–2.535	0.267
Poor collateral	1.892	1.040–3.442	0.037
Absence of MSC	2.688	1.483–4.872	0.001
Baseline occlusion location	0.777	0.516–1.171	0.229

CBS, Clot Burden Score; CBV, Cerebral Blood Volume; ASPECTS, the Alberta Stroke Program Early Computed Tomography Score; CBF, Cerebral Blood Flow; NECT, Non-Enhanced Computed Tomography; LSA, Lenticulostriate Arteries; MSC, Multi-Segment Clot.

**Table 3:** META score.

Variables	Categories	Points
**CBS**	≤6	1
	>6	0
**CBV-ASPECTS**	≤6	2
	>6	0
**Poor collateral**	Yes	1
	No	0
**Absence of MSC**	Yes	1
	No	0
**Total score**		0–5

CBS, Clot Burden Score; CBV, Cerebral Blood Volume; ASPECTS, the Alberta Stroke Program Early Computer Tomography Score; MSC, Multi-Segment Clot.

## Data Availability

The data that support the findings of this study can be made available from the corresponding author on reasonable request.
